# Flow Past a Permeable Stretching/Shrinking Sheet in a Nanofluid Using Two-Phase Model

**DOI:** 10.1371/journal.pone.0111743

**Published:** 2014-11-03

**Authors:** Khairy Zaimi, Anuar Ishak, Ioan Pop

**Affiliations:** 1 Institute of Engineering Mathematics, Universiti Malaysia Perlis, Arau, Perlis, Malaysia; 2 Centre for Modelling and Data Analysis, School of Mathematical Sciences, Faculty of Science and Technology, Universiti Kebangsaan Malaysia, UKM Bangi, Selangor, Malaysia; 3 Department of Mathematics, Babeş-Bolyai University, Cluj-Napoca, Romania; Irvine, United States of America

## Abstract

The steady two-dimensional flow and heat transfer over a stretching/shrinking sheet in a nanofluid is investigated using Buongiorno’s nanofluid model. Different from the previously published papers, in the present study we consider the case when the nanofluid particle fraction on the boundary is passively rather than actively controlled, which make the model more physically realistic. The governing partial differential equations are transformed into nonlinear ordinary differential equations by a similarity transformation, before being solved numerically by a shooting method. The effects of some governing parameters on the fluid flow and heat transfer characteristics are graphically presented and discussed. Dual solutions are found to exist in a certain range of the suction and stretching/shrinking parameters. Results also indicate that both the skin friction coefficient and the local Nusselt number increase with increasing values of the suction parameter.

## Introduction

The analysis of boundary layer flow and heat transfer of an incompressible fluid across a stretching sheet has gained attention of many researchers. Nowadays, a large amount of work has been placed to focus on this topic in view of its several applications in engineering and industrial processes. The cooling of electronic devices by the fan and nuclear reactor, polymer extrusion, wire drawing, etc are examples of such flows in engineering and industrial processes. The list of importance of flows in fluid mechanics has motivated researchers to continue the study in different types of fluid as well as in different physical aspects.

The study of flow over a stretching sheet was pioneered by Crane [1] who solved analytically the steady two-dimensional flow past a linearly stretching plate. This problem was later extended by Wang [2] to three-dimensional case. Since then many researchers have investigated various aspects of this type of flow such as Ibrahim and Shankar [3], Roşca and Pop [4], Nandy and Mahapatra [5], Kumaran et al. [6], Turkyilmazoglu [7], Ishak et al. [8]–[10], Yacob et al. [11] and Hussain et al. [12], among others. They have studied the fluid flow and some characteristics of heat transfer towards a stretching sheet in the presence of magnetic field, slip effect, convective boundary conditions, suction/injection, viscous dissipation, radiation effect and heat generation/absorption considering different types of fluid such as nanofluid, viscoelastic fluid and micropolar fluid.

In a continuation study of flow over a stretching sheet, considerable interest has been placed on fluid flow over a shrinking sheet. The study of viscous flow over a shrinking sheet with suction effect at the boundary was first investigated by Miklavčič and Wang [13]. Following this pioneering work, many papers on this topic have been published. For such problem, the movement of the sheet is in the opposite direction to that of the stretching case, and thus the flow moves towards a slot. Goldstein [14] has described the shrinking flow which is basically a backward flow. Vorticity of the shrinking sheet is not confined within a boundary layer, and the flow is unlikely to exist unless adequate suction on the boundary is imposed (Miklavčič and Wang [13]).

The “nanofluid” term was first introduced by Choi [15] to describe the mixture of nanoparticles and base fluid such as water and oil. The addition of nanoparticle into the base fluid is able to change the transport properties, flow and heat transfer capability of the liquids and indirectly increase the low thermal conductivity of the base fluid which is identified as the main obstacle in heat transfer performance. This mixture has attracted the interest of numerous researchers because of its many significant applications such as in the medical applications, transportations, microelectronics, chemical engineering, aerospace and manufacturing (Li et al. [16]). A comprehensive literature review on nanofluids has been given by Li et al. [16], Kakaç and Pramuanjaroenkij [17], Wong and De Leon [18], Saidur et al. [19], Fan and Wang [20], Jaluria et al. [21], and most recently by Mahian et al. [22]. These papers are based on the mathematical nanofluid models proposed by Khanafer [23], and Tiwari and Das [24] for the two-phase mixture containing micro-sized particles. On the other hand, one should also mention the mathematical nanofluid model proposed by Buongiorno [25] used in many papers pioneered by Nield and Kuznetsov [26], and Kuznetsov and Nield [27] for the free convection boundary layer flow along a vertical flat plate embedded in a porous medium or in a viscous fluid. In this model, the Brownian motion and thermophoresis enter to produce their effects directly into the equations expressing the conservation of energy and nanoparticles, so that the temperature and the particle density are coupled in a particular way, and that results in the thermal and concentration buoyancy effects being coupled in the same way. We also mention here the recently published papers by Rashidi et al. [28]–[30] for the nano boundary-layers over stretching surfaces, entropy generation in a steady MHD flow due to a rotating disk in a nanofluid and on the comparative numerical study of single and two phase models of nanofluid heat transfer in wavy channel.

In the present study, we aim to investigate the problem of fluid flow due to a permeable stretching/shrinking sheet in a nanofluid. The present work is based on the nanofluid model introduced by Buongiorno [25], with the new boundary condition which was very recently proposed by Kuznetsov and Nield [31], [32] and Nield and Kuznetsov [33], [34]. As it was explained in [31], the major limitation of the model used by Nield and Kuznetsov [26] was active control of nanoparticle volume fraction at the boundary. The revised model [31]–[34] has been proposed and extended it to the case when the nanofluid particle fraction on the boundary is passively rather than actively controlled. The numerical results are tabulated and shown graphically to illustrate the influence of the suction parameter and the stretching/shrinking parameter on the velocity, temperature, concentration, skin friction coefficient and the local Nusselt number. It is found that the solutions of the ordinary (similarity) differential equations have multiple (dual) solutions in a certain range of the governing parameters.

## Mathematical Formulation

**Figure 1:**
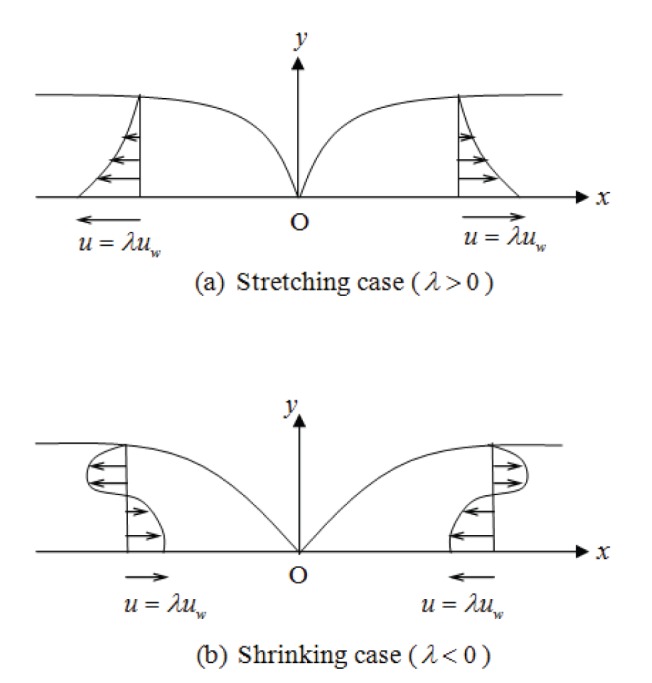
Physical model and coordinate system.



Consider a steady flow of an incompressible nanofluid in the region 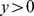 driven by a permeable stretching/shrinking surface located at 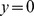 with a fixed origin 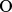 at 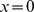 as shown in Fig. 1. The stretching/shrinking velocity 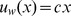 is assumed to vary linearly from the origin 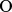, where 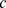 is a positive constant 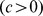. It is also assumed that the uniform temperature at the surface of the sheet is 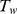, while the uniform temperature and the uniform nanofluid volume fraction far from the surface of the sheet are 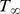 and 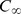, respectively. Under the above assumptions, the governing equations of the conservation of mass, momentum, thermal energy and nanoparticles equations which describe this problem can be expressed as follows (see Buongiorno [25]):

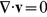
(1)




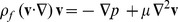
(2)




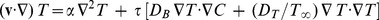
(3)




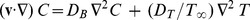
(4)


Here 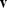 is the velocity vector, 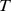 is the temperature of the nanofluid, 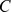 is the nanoparticle volume fraction, 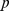 is the pressure, 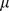 is the dynamic viscosity, 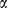 is the thermal diffusivity of the nanofluid, 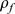 is the nanofluid density, 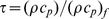 is the ratio between the effective heat capacity of the nanoparticle material and heat capacity of the fluid with 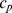 as the specific heat at constant pressure, 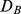 is the Brownian diffusion coefficient, 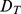 is the thermophoretic diffusion coefficient and 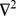 is the Laplacian operator.

By implementing the boundary layer approximations and applying the order of magnitude analysis, the governing equations (1) to (4) are transformed into the following equations:
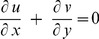
(5)




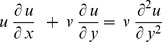
(6)




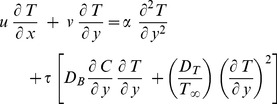
(7)




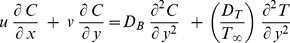
(8)where 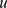 and 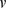 are the velocity components along the 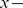 and 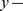 axes, and 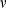 is the kinematic viscosity. Following Kuznetsov and Nield [31], [32] and Nield and Kuznetsov [33], [34] the boundary conditions of Eqs. (5)–(8) are
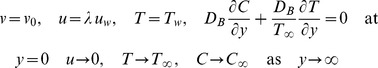
(9)where 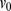 is the mass flux velocity with 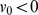 for suction and 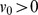 for injection. It is worth mentioning that Makinde and Aziz [35], Bachok et al. [36], [37] and Mansur and Ishak [38] employed the condition 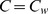 for concentration at the boundary. The major limitation of this condition is the active control of nanoparticle volume fraction at the boundary [34].

We introduce now the following transformation:
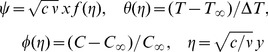
(10)where 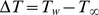, 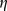 is the independent similarity variable and 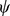 is the stream function, which is defined as 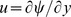 and 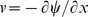 which identically satisfies the continuity equation (5). Further, 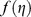 is the dimensionless stream function, 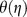 is the dimensionless temperature and 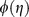 is the dimensionless nanoparticle volume fraction. Substituting (10) into Eqs. (5)–(8), we obtain the following nonlinear ordinary differential equations



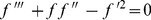
(11)

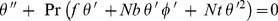
(12)




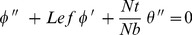
(13)and the boundary conditions (9) become



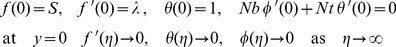
(14)Here prime denotes a differentiation with respect to 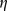, 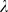 is the stretching/shrinking parameter with 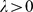 for stretching and 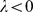 for shrinking, 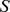 is the suction/injection parameter with 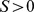 for suction and 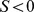 for injection, 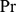 is the Prandtl number, 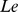 is the Lewis number, 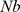 is the Brownian motion parameter and 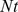 is the thermophoresis parameter, which are defined as
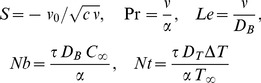
(15)


Here, 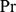, 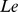, 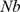 and 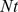 denote the Prandtl number, Lewis number, Brownian motion parameter and thermophoresis parameter, respectively.

The quantities of practical interest in this study are the skin friction coefficient 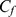 and the local Nusselt number 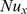, which are defined as
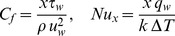
(16)where 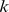 is the thermal conductivity, 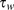 and 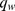 are the surface shear stress and the surface heat flux, and are defined as



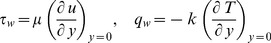
(17)Using (10), (16) and (17), we have
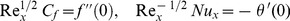
(18)where 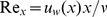 is the local Reynolds number. It should be mentioned that with the new boundary condition in (9) for 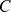, the local Sherwood number 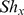, which represents the dimensionless mass flux is identically zero (see Kuznetsov and Nield [31]).

Following Fang et al. [39] it can be very easily shown that for the particular case of 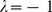 (shrinking sheet), Eq. (11) subject to the boundary conditions (14) for 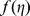 has the closed form analytical solution
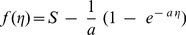
(19)where 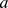 is given by



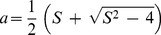
(20)with 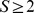. Thus 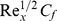 becomes
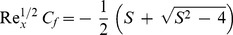
(21)


On the other hand, for the case of the stretching sheet 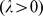, Eq. (11) with the boundary conditions (14) has the analytical solution
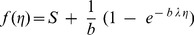
(22)with 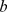 given by
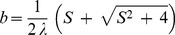
(23)for any value of 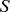. Thus, 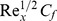 is given now by
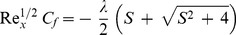
(24)


## Results and Discussion

The nonlinear ordinary differential equations (11)–(13) subject to the boundary conditions (14) were solved numerically using a shooting method with the help of Maple software [40]–[42]. This method is described in the book by Jaluria and Torrance [43] and has been successfully used by several researchers to solve the flow and heat transfer problems [44]–[47]. Dual solutions are obtained using two different initial guesses for the unknown values of 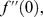 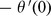 and 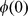 for the same value of parameter, which produce two different velocities, temperature and concentration profiles where both of them reach the far field boundary conditions (14) asymptotically. The Prandtl number 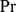 is fixed at 6.8 (water).

**Figure 2:**
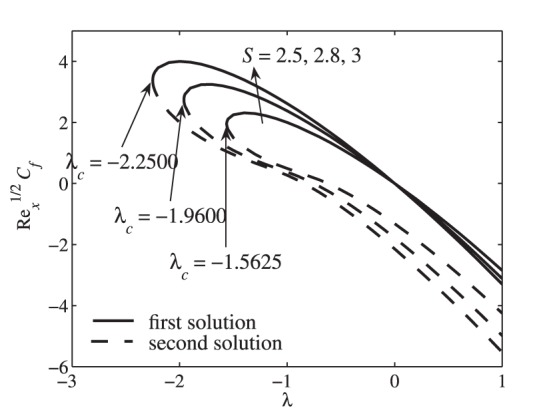
Variation of the skin friction coefficient 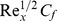 with 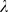 for different values of 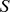 when 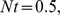 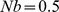, 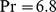 and 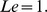

**Figure 3:**
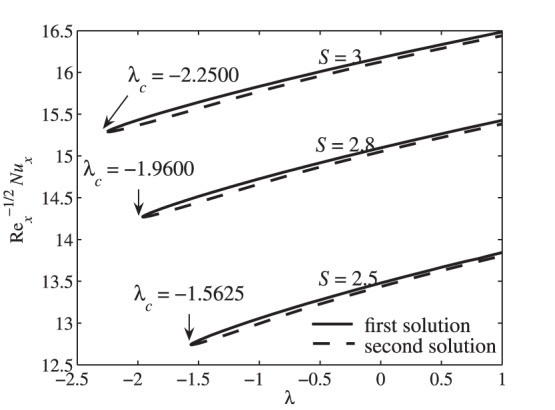
Variation of the local Nusselt number 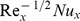 with 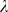 for different values of 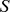 when 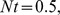 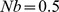, 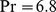 and 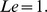

Fig. 2 presents the variation of the skin friction coefficient 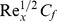 as a function of the stretching/shrinking parameter 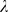 for some values of the suction parameter 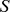, while the corresponding local Nusselt number 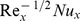 is depicted in Fig. 3. Dual solutions are found to exist for 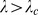, where 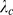 is the critical value of 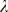 for which the solution exists. The solution is unique when 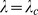 and no solution to the system of equations (11)–(14) for 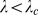. These critical values 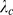 for some values of 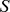 and fixed values of other parameters are presented in Table 1. The values given in Table 1 are in good agreement with the analytical result (for the flow field) obtained by Yao et al. [48], who reported that the solution domain is 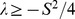. Table 2 presents the comparison of the values of 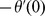 with those obtained by Grubka and Bobba [49] and Chen [50], which shows a favorable agreement. In Figs. 2 and 3, the solution terminates at the critical values 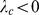 (shrinking case). However, the solution could exist for all positive values of 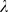 (stretching case) greater than those presented in Figs. 2 and 3. It is seen from these figures that suction widens the range of 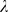 for which the similarity solution to Eqs. (11)–(13) with boundary conditions (14) exists as illustrated in Figs. 2 and 3. As in similar flow problems, the stability analysis for the multiple solutions has been done by several researchers such as Merkin [51], Weidman et al. [52], Paullet and Weidman [53], Harris et al. [54], and Postelnicu and Pop [55]. They have shown in details that only the first solution is stable and physically relevant, while the second solution is not. Thus, we expect this finding to be applicable to the present problem. Although the second solutions are deprived of physical significance, they are nevertheless of interest as far as the differential equations are concerned. Similar equations may arise in other situations where the corresponding solutions could have more realistic meaning [56].

**Table 1:** The critical values of 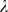, i.e. 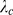, for some values of 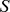 when 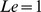,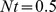, 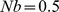, 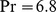.

*S*	*λ_c_*
2.5	−1.5625
2.8	−1.9600
3	−2.2500

**Table 2:** Comparison of the values of 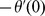 with previously published data when 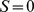, 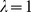 and in the absence of 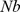 and 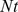.

Pr	0.72	1	3	7	10
Grubka and Bobba [49]	0.4631	0.5820	1.1652		2.3080
Chen [50]	0.46315	0.58199	1.16523	1.89537	2.30796
Present	0.463145	0.581977	1.165246	1.895403	2.308004

In Fig. 2, it is seen that the skin friction coefficient increases as 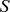 increases for the first solution. This is because of the suction effect which increases the surface shear stress and in turn increases the velocity gradient at the surface 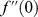. Consequently, the skin friction coefficient increases with increasing the suction effect.

Fig. 3 demonstrates the variation of the local Nusselt number 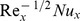, which represents the heat transfer rate at the surface for some values of the suction parameter *S*. It clearly indicates that the local Nusselt number increases as 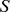 increases for both first and second solutions. This observation occurs due to the suction effect which increases the surface shear stress and in consequence increases the heat transfer rate at the surface. It is also noticed that the local Nusselt number increases (in absolute sense) as 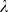 increases for both solutions. Thus, the heat transfer rate at the surface increases with increasing the stretching effect.

**Figure 4:**
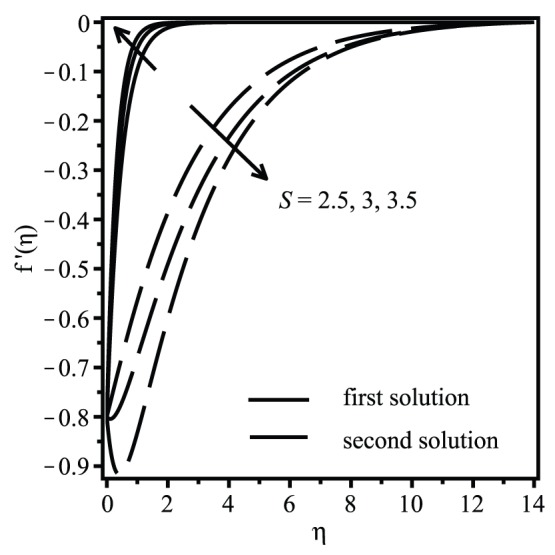
Effect of the suction parameter 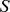 on the velocity profiles 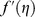 when 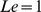,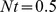, 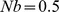, 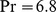 and 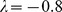 (shrinking case).

**Figure 5:**
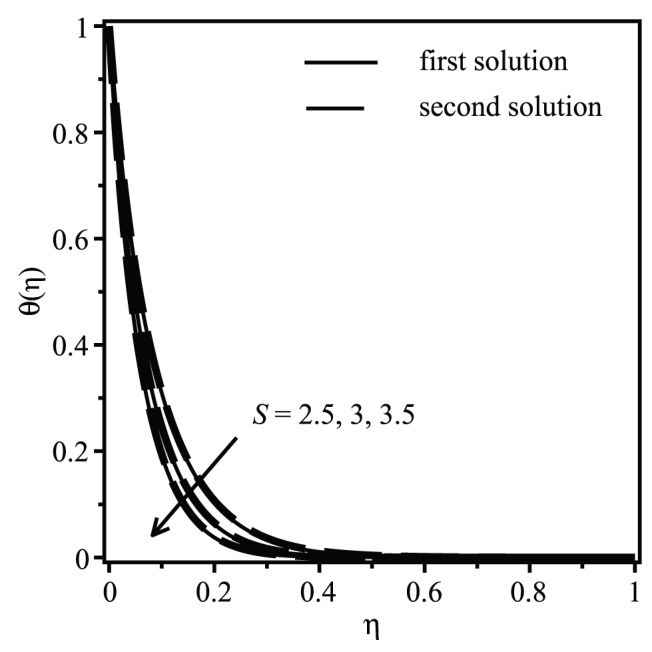
Effect of the suction parameter 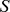 on the temperature profiles 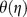 when 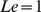, 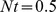, 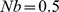, 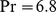 and 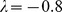 (shrinking case).

Figs. 4 and 5 are depicted to show the effect of suction on the velocity and temperature for the shrinking case 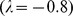. It is seen that there exist two different profiles for a particular value of 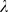, i.e. 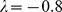, but with different shapes and boundary layer thicknesses, which support the existence of dual solutions presented in Figs. 2 and 3. For the first solution, which we expect to be the physically realizable solution, Fig. 4 shows that increasing suction is to decrease the fluid velocity inside the boundary layer and in consequence increase the velocity gradient at the surface. Increasing suction is to increase the skin friction coefficient which results in increasing manner of the heat transfer rate at the surface. This can be seen in Fig. 5 where the temperature gradient at the surface increases as suction increases. This observation is consistent with the results presented in Fig. 3.

The samples of velocity and temperature profiles for selected values of parameters displayed in Figs. 4 and 5 show that the infinity boundary conditions (14) are satisfied asymptotically, which supports the validity of the numerical results obtained, besides supporting the existence of dual solutions presented in Figs. 2 and 3.

We mention to this end the very interesting papers by Turkyilmazoglu [57], [58] on multiple solutions of hydromagnetic permeable flow and heat for viscoelastic fluid past a shrinking sheet and on the analytic heat and mass transfer of the mixed hydrodynamic/thermal slip MHD viscous flow over a stretching sheet.

## Conclusions

The boundary layer flow and heat transfer due to a permeable stretching/shrinking sheet in a nanofluid was considered, for the situation when the nanofluid particle fraction on the boundary is passively rather than actively controlled. The effects of the suction parameter 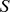 and the stretching/shrinking parameter 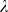 on the skin friction coefficient and the local Nusselt number are graphically illustrated and analyzed. It was observed that suction increases the skin friction coefficient (in absolute sense) and the local Nusselt number. The heat transfer rate at the surface increases with increasing the stretching effect. Dual solutions were found to exist in a certain range of 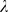 for both stretching and shrinking cases.

## References

[1] Crane LJ (1970) Flow past a stretching plate. Z. angew. Math. Phy. 21: 645–647.

[2] Wang CW (1984) The three-dimensional flow due to a stretching flat surface. Phys. Fluids 27: 1915–1917.

[3] Ibrahim W, Shankar B (2013) MHD boundary layer flow and heat transfer of a nanofluid past a permeable stretching sheet with velocity, thermal and solutal slip boundary conditions. Comp. Fluids 75: 1–10.

[4] Roşca AV, Pop I (2013) Flow and heat transfer over a vertical permeable stretching/shrinking sheet with a second order slip. Int. J. Heat and Mass Transfer 60: 355–364.

[5] Nandy SK, Mahapatra TR (2013) Effects of slip and heat generation/absorption on MHD stagnation flow of nanofluid past a stretching/shrinking surface with convective boundary conditions. Int. J. Heat and Mass Transfer 64: 1091–1100.

[6] Kumaran V, Banerjee AK, Vanav Kumar A, Pop I (2011) Unsteady MHD flow and heat transfer with viscous dissipation past a stretching sheet, Int. Comm. Heat and Mass Transfer 38: 335–339.

[7] Turkyilmazoglu M (2011) Multiple solutions of heat and mass transfer of MHD slip flow for the viscoelastic fluid over a stretching sheet. Int. J. of Thermal Sciences 50: 2264–2276.

[8] Ishak A, Nazar R, Pop I (2006) Unsteady mixed convection boundary layer flow due to a stretching vertical surface. Arab. J. Sci. Eng. 31: 165–182.

[9] Ishak A, Nazar R, Pop I (2008) MHD boundary-layer flow due to a moving extensible surface. J. Eng. Math. 62: 23–33.

[10] Ishak A, Nazar R, Pop I (2008) Magnetohydrodynamic (MHD) flow and heat transfer due to a stretching cylinder. Energy Conversion Management 49: 3265–3269.

[11] Yacob NA, Ishak A, Pop I, Vajravelu K (2011) Boundary layer flow past a stretching/shrinking surface beneath an external uniform shear flow with a convective surface boundary condition in a nanofluid. Nanoscale Research Letter 6: Article Number 314. 10.1186/1556-276X-6-314 PMC3211401 21711841

[12] Hussain M, Ashraf M, Nadeem S, Khan M (2013) Radiation effects on the thermal boundary layer flow of a micropolar fluid towards a permeable stretching sheet. J. Franklin Inst. 350: 194–210.

[13] Miklavčič M, Wang CY (2006) Viscous flow due to a shrinking sheet. Quart. Appl. Math. 64: 283–290.

[14] Goldstein J (1965) On backward boundary layers and flow in converging passages. J. Fluid Mech. 21: 33–45.

[15] Choi SUS (1995) Enhancing thermal conductivity of fluids with nanoparticles, Developments and Applications of Non-Newtonian Flows. FED-vol.231/MD-vol. 66: 99–105.

[16] Li Y, Zhou J, Tung S, Schneider E, Xi S (2009) A review on development of nanofluid preparation and characterization. Powder Tech. 196: 89–101.

[17] Kakaç S, Pramuanjaroenkij A (2009) Review of convective heat transfer enhancement with nanofluids. Int. J. Heat Mass Transfer 52: 3187–3196.

[18] Wong KV, De Leon O (2010) Applications of nanofluids: current and future. Adv. Mech. Eng. 2010: Article ID 519659, 11 pages.

[19] Saidur R, Leong KY, Mohammad HA (2011) A review on applications and challenges of nanofluids. Renew. Sust. Ener. Rev. 15: 1646–1668.

[20] Fan J, Wang L (2011) Review of heat conduction in nanofluids. ASME J. Heat Transfer 133: Article ID 040801.

[21] Jaluria Y, Manca O, Poulikakos D, Vafai K, Wang L (2012) Heat transfer in nanofluids. Adv. Mech. Eng. 2012: Article ID 972973, 2 pages.

[22] Mahian O, Kianifar A, Kalogirou SA, Pop I, Wongwises S (2013) A review of the applications of nanofluids in solar energy. Int. J. Heat Mass Transfer 57: 582–594.

[23] Khanafer K, Vafai K, Lightstone M (2003) Buoyancy-driven heat transfer enhancement in a two-dimensional enclosure utilizing nanofluids. Int. J. Heat Mass Transfer 46: 3639–3653.

[24] Tiwari RK, Das MK (2007) Heat transfer augmentation in a two-sided lid-driven differentially heated square cavity utilizing nanofluids. Int. J. Heat Mass Transfer 50: 2002–2018.

[25] Buongiorno J (2006) Convective transport in nanofluids. ASME J. Heat Transfer 128: 240–250.

[26] Nield DA, Kuznetsov AV (2009) The Cheng-Minkowycz problem for natural convective boundary layer flow in a porous medium saturated by a nanofluid. Int. J. Heat Mass Transfer 52: 5792–5795.

[27] Kuznetsov AV, Nield DA (2010) Natural convective boundary-layer flow of a nanofluid past a vertical plate. Int. J. Therm. Sci. 49: 243–247.

[28] Rashidi MM, Erfani E (2011) The modified differential transform method for investigating nano boundary-layers over stretching surfaces. Int. J. Num. Methods Heat Fluid Flow 21: 864–883.

[29] Rashidi MM, Abelman S, Freidoonimehr N (2013) Entropy generation in steady MHD flow due to a rotating porous disk in a nanofluid. Int. J. Heat Mass Transfer 62: 515–525.

[30] Rashid MM, Hosseini A, Pop I, Kumar S, Freidoonimehr N (2014) Comparative numerical study of single and two phase models of nanofluid heat transfer in wavy channel. Appl. Math. Mech.-Engl. Ed. 35: 1–18.

[31] Kuznetsov AV, Nield DA (2013) The Cheng-Minkowycz problem for natural convective boundary layer flow in a porous medium saturated by a nanofluid: a revised model. Int. J. Heat Mass Transfer 65: 682–685.

[32] Kuznetsov AV, Nield DA (2014) Natural convective boundary-layer flow of a nanofluid past a vertical plate: A revised model. Int. J. Thermal Sci. 77 (2014) 126–129.

[33] Nield DA, Kuznetsov AV (2014) Thermal instability in a porous medium layer saturated by a nanofluid: A revised model. Int. J. Heat Mass Transfer 68 (2014) 211–214.

[34] Nield DA, Kuznetsov AV (2014) The onset of convection in a horizontal nanofluid layer of finite depth: A revised model. Int. J. Heat Mass Transfer 77 (2014) 915–918.

[35] Makinde OD, Aziz A (2011) Boundary layer flow of a nanofluid past a stretching sheet with a convective boundary condition. Int. J. Thermal Sci. 50: 1326–1332.

[36] Bachok N, Ishak A, Pop I (2010) Boundary-layer flow of nanofluids over a moving surface in a flowing fluid. Int. J. Thermal Sci. 49: 1663–1668.

[37] Bachok N, Ishak A, Pop I (2012) Unsteady boundary-layer flow and heat transfer of a nanofluid over a permeable stretching/shrinking sheet. Int. J. Heat Mass Transfer 55: 2102–2109.

[38] Mansur S, Ishak A (2013) The flow and heat transfer of a nanofluid past a stretching/shrinking sheet with a convective boundary condition. Abstract Appl. Anal. 2013: Article ID 350647.

[39] Fang T, Yao S, Zhang J, Aziz A (2010) Viscous flow over a shrinking sheet with a second order slip flow model. Commun. Nonlinear Sci. Numer. Simulat. 15: 1831–1842.

[40] Heck A (2003) Introduction to Maple. Springer, New York.

[41] Abell ML, Braselton JB (2005) Maple by Example. Elsevier, Amsterdam.

[42] Richards D (2002) Advanced Mathematical Methods with Maple. Cambridge University Press, Cambridge.

[43] Jaluria Y, Torrance KE (2003) Computational Heat Transfer, Taylor & Francis, New York.

[44] Rohni AM, Ahmad S, Pop I (2012) Note on Cortell’s non-linearly stretching permeable sheet. Int. J. Heat Mass Transfer 55: 5846–5852.

[45] Zaimi K, Ishak A, Pop I (2012) Boundary layer flow and heat transfer past a permeable shrinking sheet in a nanofluid with radiation effect. Adv. Mech. Eng. 2012: Article number 340354.

[46] Bhattacharyya K, Mukhopadhyay S, Layek GC, Pop I (2012) Effects of thermal radiation on micropolar fluid flow and heat transfer over a porous shrinking sheet. Int. J. Heat Mass Transfer 55: 2945–2952.

[47] Bachok N, Ishak A, Pop I (2013) Mixed convection boundary layer flow over a moving vertical flat plate in an external fluid flow with viscous dissipation effect. PLoS ONE 8: Article ID e60766. 10.1371/journal.pone.0060766 PMC3618105 23577156

[48] Yao S, Fang T, Zhong Y (2011) Heat transfer of a generalized stretching/shrinking wall problem with convective boundary conditions. Commun. Nonlinear Sci. Numer. Simulat. 16: 752–760.

[49] Grubka LJ; Bobba KM (1985) Heat transfer characteristics of a continuous, stretching surface with variable temperature. ASME J. Heat Trans 107: 248–250.

[50] Chen CH (1998) Laminar mixed convection adjacent to vertical, continuously stretching sheets. Heat Mass Transfer 33: 471–476.

[51] Merkin JH (1985) On dual solutions occurring in mixed convection in a porous medium. J. Eng. Math. 20: 171–179.

[52] Weidman PD, Kubitschek DG, Davis AMJ (2006) The effect of transpiration on selfsimilar boundary layer flow over moving surfaces. Int. J. Eng. Sci. 44: 730–737.

[53] Paullet J, Weidman PD (2007) Analysis of stagnation point flow towards a stretching sheet. Int. J. Nonlinear Mech. 42, 1084–1091.

[54] Harris SD, Ingham DB, Pop I (2009) Mixed convection boundary layer flow near the stagnation point on a vertical surface in a porous medium: brinkman model with slip. Transp. Porous Media 77: 267–285.

[55] Postelnicu A, Pop I (2011) Falkner-Skan boundary layer flow of a power-law fluid past a stretching wedge. Appl. Math. Comput. 217, 4359–4368.

[56] Ridha A (1996) Aiding flows non-unique similarity solutions of mixed-convection boundary-layer equations. Z. angew. Math. Phys. 47: 341–352.

[57] Turkyilmazoglu M (2011) Multiple solutions of hydromagnetic permeable flow and heat for viscoelastic fluid. J. Thermophys. Heat Transfer 4: 595–605.

[58] Turkyilmazoglu M (2011) Analytic heat and mass transfer of the mixed hydrodynamic/thermal slip MHD viscous flow over a stretching sheet. Int. J. Mechanical Sci. 53: 886–896.

